# Novel Synthesis of Polystyrenesulfonate@AC Based on Olive Tree Leaves Biomass for the Photo-Degradation of Methylene Blue from Aqueous Solution

**DOI:** 10.3390/polym16233321

**Published:** 2024-11-27

**Authors:** Ibrahim Hotan Alsohaimi

**Affiliations:** Chemistry Department, College of Science, Jouf University, Sakaka 2014, Saudi Arabia; ehalshaimi@ju.edu.sa

**Keywords:** photo-degradation, methylene blue, photocatalyst, activated carbon

## Abstract

Water pollution poses significant environmental challenges, particularly from dyes used in various industrial processes. Effective removal methods are essential to mitigate their impact on aquatic environments. Activated carbon (AC) is widely used for its adsorption properties, and further modifications can enhance its efficiency. In this study, we developed polystyrene sulfonate-modified activated carbon (AC@PSS) using a facile and efficient method to improve the photo-degradation of methylene blue (MB) in aquatic environments. The modification enhanced the activated carbon’s surface features and adsorption, improving its photocatalytic activity. The photocatalysts were characterized using XRD, SEM, FTIR, and TGA. Based on Tauc’s equation, the band gap value of AC@PSS was 4.0 eV. The photocatalytic efficacy of the AC@PSS catalyst was assessed by studying the degradation of MB dye under UV-rich solar irradiation. The influence of various variables on the photo-degradation of MB dye such as pH (2–12), reaction time (0–160 min), catalyst dosage (20–80 mg), and dye concentration (10–300 mg/L) was investigated. The AC@PSS catalyst demonstrated impressive degradation efficacy for MB dye of 98% in 160 min at pH 11, a temperature of 25 °C, a catalyst dose of 60 mg, and initial MB content of 10 mg/L. The superior performance of the AC@PSS catalyst could be due to the effective separation of photogenerated electron holes. Accordingly, the photo-degradation of MB is affected by the photo-produced radical ^•^OH. Finally, we conclude that synthesizing AC@PSS is highly effective for the degradation of MB dye.

## 1. Introduction

Organic dyes are one of the major contaminants that result from the textiles, pharmaceutical, plastic, rubber, paper, and paint industries that pose a threat to human health. Approximately 10,000 textile pigments are produced annually, and more than 100 tons are released into the water environment [1,2]. Organic dyes are very stable and slow to degrade and can remain in water bodies for long periods, posing environmental threats. MB is a cationic dye extensively used in the textile industry to dye silk, linen, and wool fabrics [3]. It can have serious health effects, including eye damage, vomiting, skin irritation, and altered mental status [4]. Therefore, it is essential to eliminate MB from contaminated water before discharging it into the water environment. There are various methods to remove dye contaminants, including photocatalysis, oxidation processes, zonation, nanofiltration microbial treatment, coagulation, and surface adsorption. Adsorption and photo-catalysis are an effective method for the elimination and degradation of toxic dyes from wastewater. Although adsorption is a widespread method used to remove pollutants, the photocatalytic method is preferred for organic pollutants with aromatic rings and photosensitivity [5]. Photocatalysis uses sunlight in the existence of semiconductors to generate free radical species (^•^OH and ^•^O_2_^−^) and electron holes (h^+^) to degrade the dyes and pharmaceutical pollutants into water and carbon dioxide.

Metallic nanoparticles (NPs) significantly enhance photocatalytic activity by serving as electron traps, reducing charge recombination, and facilitating the generation of reactive oxygen species for efficient pollutant degradation. Agnieszka Sidorowicz et al. [6] developed a sustainable method to synthesize silver nanoparticles (Ag NPs) using *Spirulina platensis* extracts. The Ag NPs demonstrated excellent photocatalytic activity, achieving 81.9% degradation of brilliant blue R dye under visible light in alkaline conditions, with a k_app_ value of 0.00595 min^−1^ after 90 min. Furthermore, many metal oxides, such as titanium dioxide, have been applied as photocatalysts to degrade different contaminants from aqueous solutions [7,8]. Activated carbon (AC) is widely recognized as one of the most effective adsorbents for water purification because of its large porosity, internal surface area, and variety of oxygenated functional groups [8]. In addition, activated carbon is used as a photocatalyst support to accelerate the rate of pollutant degradation compared to bare photocatalysts [9]. AC aids in charge separation and facilitates the reaction between photogenerated electrons and adsorbed oxygen, which decreases electron–hole pair recombination [10]. Therefore, different activated carbon-supported photocatalysts, such as TiO_2_/AC [11], Al_2_O_3_/AC [12], ZnO/AC [13], AC-WO3 [14], and MoS_2_/AC [15], are used for eliminating various pollutants. Bhavsar et al. prepared activated carbon-loaded CdS to degrade methylene blue. They found that the AC-CdS assists in efficient charge separation, leading to enhanced photocatalytic performance [10]. Van Hung and co-workers found that the photo-degradation of MB using ZnO/AC was 9.79 times faster compared to pure ZnO [16]. Activated carbon AC has many functional moieties, such as –COOH, –C = O, and OH groups, on its surface. Modifying activated carbon with functional groups of photocatalytic materials can affect the electronic properties, surface chemistry, and performance of the photocatalytic system [17].

The main aim of this article is to incorporate polystyrene sulfonate o activated carbon using a simple method as a novel photocatalyst for the degradation of MB dye from aquatic environments. Therefore, this study aimed firstly to prepare activated carbon derived from olive leaf via chemical activation with KOH as an activating agent. Secondly, polystyrene sulfonate on activated carbon was introduced using a facial method. Thirdly, AC@PSS was characterized using various techniques such as FTIR, XRD, TGA, and SEM. Fourth, to study the ability of novel photocatalysts to degrade MB from aquatic environments. The impact of various parameters on the photo-degradation of MB dye such as pH, dosage, reaction time, and catalyst concentration was studied. The modification of activated carbon (AC) with polystyrene sulfonate (PSS) introduces unique structural and electronic properties, contributing to its photocatalytic and adsorptive capabilities. Polystyrene sulfonate is known for its high ion exchange capacity and ability to enhance electron mobility, which can reduce the recombination of photogenerated electron–hole pairs on the composite’s surface. In this composite system, activated carbon acts as a porous adsorbent with a high surface area, providing numerous active sites for pollutant adsorption. PSS functionalization improves the surface’s hydrophilicity and charge transfer properties. The synergistic interaction between AC and PSS promotes enhanced adsorption, followed by photocatalytic degradation. In particular, the electron-donating sulfonate groups can aid in the separation of photogenerated charges, preventing recombination and facilitating the generation of reactive oxygen species (ROS) such as hydroxyl radicals (^•^OH) and superoxide radicals (O_2_^−•^). This hybrid mechanism of adsorption-assisted photo-degradation makes AC@PSS a promising candidate for environmental remediation applications, as the adsorbed methylene blue molecules are more effectively degraded under UV-rich solar irradiation.

## 2. Experiment

### 2.1. Materials

Methylene blue (MB, Reag. Ph Eur, Darmstadt, Germany), N, N-dimethylformamide (DMF, ≥99.5%, Sigma Aldrich Co., St. Louis, MO, USA), Styrene (St, ≥99%, Sigma-Aldrich Co., USA), chloroacetyl chloride, (CAC, Sigma Aldrich Co., USA), potassium hydroxide, (DMF, ≥99.5%, Sigma Aldrich Co., USA), chloroform (CHCl_3_, 99.0–99.4%, BDH Chemicals Ltd., Poole, UK), Nitric acid (HNO3, 70%, BDH Chemicals Ltd., Poole, UK), and sulfuric acid (H_2_SO_4_, 70%, BDH Chemicals Ltd., Poole, UK) were utilized in the experiments. Deionized water was utilized in all experiments.

### 2.2. Characterization

The prepared materials (AC and AC@PSS) were characterized through the application of scanning electron microscopy (SEM, QUANTA 250 FEI; Hillsboro, OR, USA), an X-ray diffractometer (D/Max2500VB2+/Pc, Shimadzu Company, Kyoto, Japan), and Fourier-transform infrared (Shimadzu IR Tracer—100) thermogravimetric analysis (TGA—51 Shimadzu TGA analyzer).

### 2.3. Synthesis of Polystyrene Sulfonate@ Activated Carbon

#### 2.3.1. Preparation of Olive Leaves Powder

Olive leaves were collected from olive trees in the Jouf Region, Saudi Arabia. The olive leaves were soaked from the roots to the branches and then washed well with distilled water three times. Next, the olive leaves were dried at 110 °C and then ground and sieved to 75 microns. Olive leaves powder was utilized as the starting material for the fabrication of activated carbon using a chemical modification approach using KOH as an activation reagent.

#### 2.3.2. Preparation of Olive Oil-Based Activated Carbon

The chemical activation process of the olive oil powder was carried out using the following procedures: typically, 5 g of the ground olive leaf powder was electrically heated in the tube furnace under N_2_ at a heating rate (3 °C min^−1^) of 25 to 500 °C for 3 h. Then, the carbonized olive leaves were soaked into the activating agent solution (KOH) for 3 h. After that, the impregnated carbon was dried at 110 °C for 18 h. After that, the dried impregnated carbon was carbonized in an inert atmosphere (N_2_) at high temperatures (700 °C) for activation. Finally, the activated carbon was rinsed many times with deionized water to remove excess chemicals and neutralize it and then dried at 110 °C for 24 h.

#### 2.3.3. Synthesis of the AC@PSS Composite

**Figure 1:**
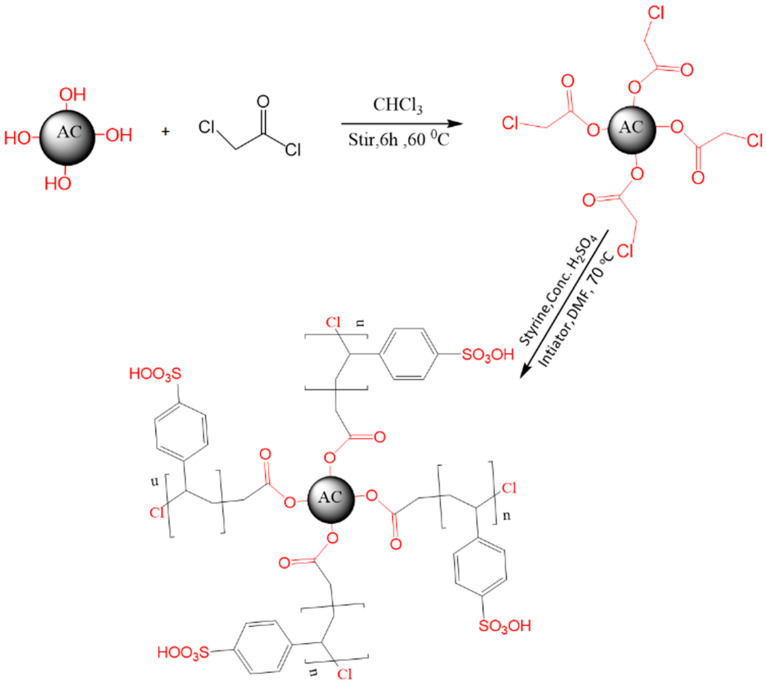
Schematic diagram of the synthesis of the AC@PSS composite.

Polystyrene sulfonate-incorporated activated carbon was prepared via a novel and simple method. A mixture of carbon olive ash (1 gm) and 5 mL chloroacetyl chloride in 20 mL chloroform was heated under reflux for 6 h. The resulting precipitate was then collected and dried to afford the chloride derivative in good yield. After that, 1 g of chloride derivative, 7 mL of styrene, 5 mL of concentration sulfuric acid, and 1 g of initiator in 20 mL dimethylformamide were heated at 70 °C for 8 h. Then, the resulting suspension was poured into an ice/water mixture and stirred to obtain the solid residue. Finally, the sulfonate-incorporated activated carbon was filtered off and dried at 50 °C for 24 h (Figure 1).

### 2.4. Photocatalytic Tests

Photocatalytic remediation of MB dye was carried out under UV-rich solar irradiation. First, 60 mg of AC@PSS was added to 25 mL of 10 mg/L solution of MB. The prepared solution was kept in the dark for 90 min to ensure uptake equilibrium before exposure to UV-rich solar irradiation. At a fixed time, 3 mL of the reaction mixture was collected and centrifuged to isolate the catalyst. Then, the solution was examined using a UV-Vis spectrophotometer at 660 nm (max). Photocatalytic degradation (%) was estimated using Equation (1):

(1) $$\mathrm{photocatalytic}\;\mathrm{degradation}\;\left(\%\right)=\frac{A_{o}-A_{t}}{A_{o}}\times 100=\frac{C_{o}-C_{t}}{C_{o}}\times 100$$

where C_o_, A_o_, C_t_, and A_t_ refer to the MB concentration and absorbance before and after degradation. Various parameters, such as pH (2–12) reaction time (0–160 min), catalyst dosage (20–80 mg), and the initial concentration (10–300 mg/L), were studied.

## 3. Results and Discussion

### 3.1. Characterization of Adsorbents

**Figure 2:**
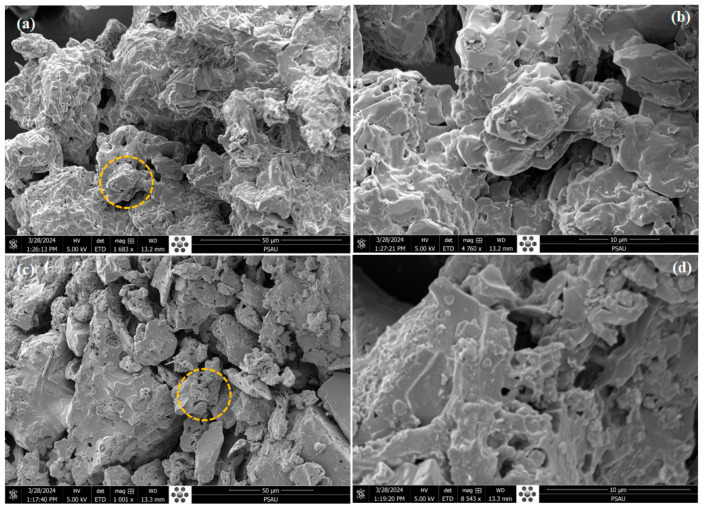
(**a**,**b**) SEM images of AC and (**c**,**d**) AC@PSS composites.

The SEM images provide detailed insights into the morphological differences between the activated carbon (AC) and the polystyrene sulfonate-modified activated carbon (AC@PSS) composites. In the SEM images of AC (Figure 2a,b), the surface morphology shows irregular and rough textures, with distinct pore structures visible. The images reveal a highly porous and amorphous structure typical of activated carbon, crucial for its adsorption properties. In contrast, the SEM images of the AC@PSS composite (Figure 2c,d) display significant modifications to the surface morphology. The polystyrene sulfonate (PSS) modification appears to have coated the surface of the activated carbon, resulting in a smoother and more uniform texture. The pores are still visible but seem more homogenously distributed and less pronounced than the unmodified AC. This suggests that the PSS coating has filled some pores or formed a thin layer over the surface, which could enhance the composite’s adsorption and photocatalytic properties. These morphological changes, observed in the SEM images, indicate successful modification of AC with PSS. The smoother surface and altered pore structure of AC@PSS will likely contribute to improved interaction with methylene blue molecules, thereby enhancing the composite’s overall performance in photo-degradation applications.

**Figure 3:**
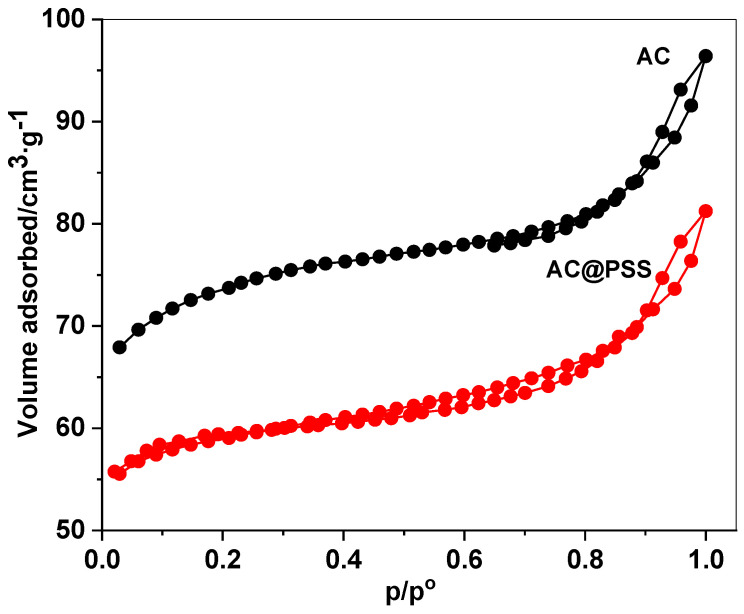
N_2_ isotherms of AC and AC@PSS composites.

The nitrogen adsorption–desorption isotherms of activated carbon (AC) and polystyrene sulfonate-modified activated carbon (AC@PSS) (Figure 3) reveal essential insights into their pore structures and surface characteristics. Both materials exhibit a Type I/IV hybrid isotherm, indicating a microporous structure with contributions from mesoporosity. The unmodified AC demonstrates high nitrogen uptake at low relative pressures (P/Po < 0.1). This reflects its well-developed microporous network and significant surface area, which is critical for adsorbing reactants and enhancing photocatalytic activity. Upon modification with polystyrene sulfonate (PSS), a reduction in nitrogen uptake is observed, particularly in the microporous region, suggesting partial pore blockage due to the coating. However, a slight increase in adsorption at intermediate relative pressures (P/Po = 0.3–0.8) indicates the introduction of mesoporosity, which may improve diffusion and facilitate access to active sites during photocatalytic degradation. The specific surface area of AC was measured at 60.25 m^2^/g, which decreased to 50.15 m^2^/g after modification with polystyrene sulfonate. This reduction in surface area can be attributed to partial pore blocking caused by the PSS coating. The total pore volume showed the same trend, decreasing from 0.1205 cm^3^/g for AC to 0.1003 cm^3^/g for AC@PSS. These changes in surface area and pore volume reflect the structural alterations induced by the PSS coating, which may play a crucial role in enhancing diffusion and adsorption during photocatalytic applications.

**Figure 4:**
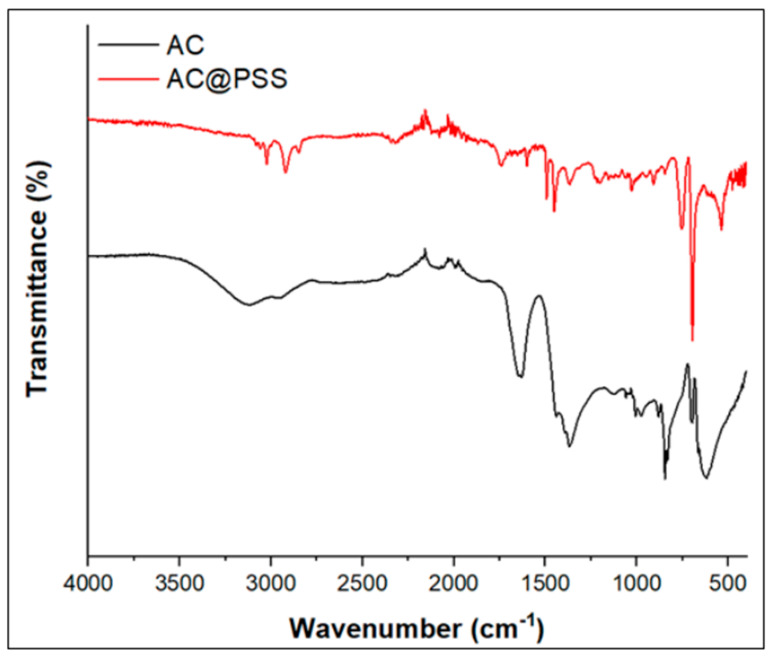
FTIR spectrum of AC and AC@PSS composites.

Figure 4 displays the FTIR spectrum of AC and AC@PSS. The FTIR spectra of AC showed various bands at 3123, 2945, 1635, 1436, 1361, 1119, 845, and 613 cm^−1^ attributed to the different molecules such as lignin, hemicellulose, and cellulose present in olive leaves [18,19,20]. In detail, the band at 3123 and 2945 cm^−1^ is related to the stretching mode of ν(–OH) and ν(–C–H), respectively [19,21]. The bands at 1635 cm^−1^ are assigned to the stretching vibration of ν(C = O) in hemicellulose and lignin [19]. The peaks at 1436 and 1361 cm^−1^ are owing to the (C = C) stretching in aromatic rings and C–H asymmetric deformation, respectively [19,22]. The band in the range (1128–1009 cm^−1^) is due to the C–O vibration [19,22]. The bands at 845 and 613 cm^−1^ are assigned to the ring C–H out-of-plane bending [18]. In the spectra of the AC@PSS, different bands were observed at 3054, 1600, and 1491 cm^−1^, which are assigned to unsaturated aromatic ν(–C-H) and (C = C) stretching in aromatic rings, respectively [23]. The bands at 2920 and 2850 cm^−1^ are due to the asymmetrical and symmetrical ν(C–H) methylene (CH_2_) group, respectively [23]. The peaks at 754, 692, and 533 cm^–1^ describe various substitutions of the benzene ring of the polystyrene [23]. The characteristic band at 1739 cm^−1^ is because of the ester group. The characteristic bands in the range of 1002–1029 cm^−1^ are attributed to the symmetric stretching of the S-O of the SO_3_H moiety [24,25,26]. All of these findings indicate the development of polystyrene sulfonate on the activated carbon.

**Figure 5:**
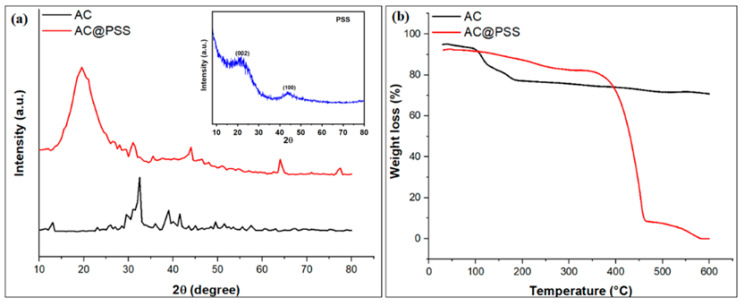
(**a**) XRD patterns of AC and AC@PSS composites (the inset is the XRD pattern of PSS) and (**b**) TGA profiles of AC and AC@PSS composites.

Figure 5a displays the XRD pattern of AC and AC@PSS. The XRD pattern of AC showed various diffraction peaks at 2θ = 32.12° (111), 39.04° (107), 43.71° (207), 49.62° (206), and 57.54° (304), which are attributed to the hexagonal crystal-structure of the carbon-based material and agree well with JCPDS: 721,616 [27,28,29]. The XRD pattern of pure polystyrene sulfonate (PSS) (Figure 5a) reveals its amorphous nature with distinct broad peaks. The broad diffraction feature around 2θ ≈ 20° corresponds to the (002) plane, characteristic of the disordered arrangement of polymer chains, indicating the amorphous structure of PSS. Another weak diffraction peak observed around 2θ ≈ 40° is attributed to the (100) plane, which may result from some local ordering or short-range interactions within the polymer matrix. The absence of sharp peaks confirms the lack of significant crystallinity in the material, which is typical for polymers like PSS. This amorphous nature contributes to its flexibility and high surface area, which is beneficial for various applications, including surface modification and adsorption processes. For the AC@PSS composite, the broad peak was observed at 2θ = 19.92°, belonging to the polystyrene [23], as well as small peaks at 2θ = 30.98°, 35.52°, 44.11°, 64.07°, and 77.45° for the crystal structure of activated carbon. These results confirmed that the polystyrene sulfonate has been impregnated onto the activated carbon structure. The mean crystalline size (D) was estimated by employing Scherer’s equation;

(2) $$D=\frac{K\lambda }{\beta cos\theta }$$

Here, K denotes the Scherer constant, taken as 0.9, λ is the X-ray wavelength (Cu Kα1 = 1.542 Å), β is the full width at half maximum of the diffraction peak, and θ is the angle. The average particle size of AC@PSS was calculated to be 16 nm.

The Thermogravimetric Analysis (TGA) curves provide insights into the thermal stability and decomposition behavior of the activated carbon (AC) and polystyrene sulfonate-modified activated carbon (AC@PSS) composites (Figure 5b). For the AC (black curve), the TGA profile shows a relatively stable weight loss up to around 600 °C, indicating good thermal stability. The significant weight loss observed for activated carbon (AC) between 100–200 °C in the thermogravimetric analysis (TGA) results can be attributed primarily to the loss of physically adsorbed water and volatile organic compounds. Activated carbon typically has a porous structure with a high surface area, which allows it to adsorb water molecules and other volatile substances from the environment. These adsorbed molecules are released upon heating, resulting in the observed weight loss. This temperature range is insufficient to break down carbonaceous materials or decompose chemically bound groups, confirming that the weight loss is due to the desorption of moisture and low-boiling-point volatiles rather than structural degradation. The phenomenon is typical for materials with a porous nature like AC, highlighting its hydrophilic surface properties to some extent. The gradual weight loss observed up to 600 °C indicates the decomposition of the organic components and the oxidation of the carbon material. In contrast, the TGA profile of the AC@PSS composite (red curve) shows a different thermal behavior. The initial weight loss below 100 °C is related to the moisture and volatile substances’ evaporation. Significant weight loss occurs between approximately 300 °C and 450 °C, which is due to the degradation of the polystyrene sulfonate (PSS) coating. This indicates that the PSS modification introduces a distinct thermal degradation phase compared to the unmodified AC. The TGA curve of AC@PSS exhibits more pronounced weight loss in the 300–450 °C range, followed by leveling off, suggesting the complete degradation of the PSS layer. This significant weight loss indicates the PSS’s presence and subsequent thermal decomposition, confirming its successful incorporation into the AC matrix. Overall, the TGA analysis highlights the differences in thermal stability between the unmodified AC and the AC@PSS composite, with the latter showing an additional decomposition phase due to the presence of the PSS. This information is crucial for understanding the thermal behavior and stability of the modified material, which can influence its performance in various applications, including the photo-degradation of methylene blue from aqueous solutions.

**Figure 6:**
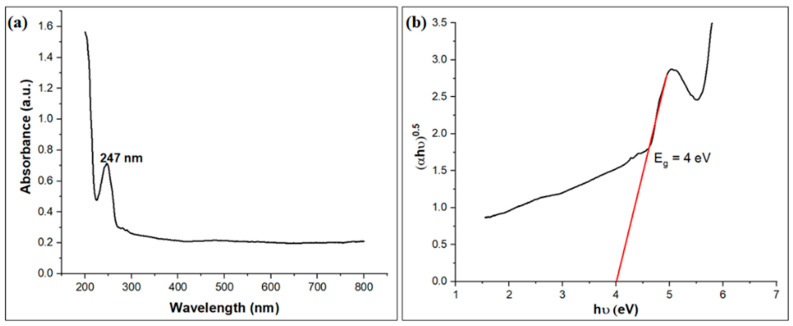
(**a**) UV–vis absorption spectra and (**b**) Tauc’s profile for the band gap assessment of the AC@PSS composite.

The UV–vis absorption spectra and Tauc’s profile for the band gap assessment provide valuable insights into the optical characteristics of the AC@PSS composite. Figure 6a shows the UV–vis absorption spectrum of the AC@PSS composite. The spectrum displays a prominent absorption peak at around 247 nm, indicative of the π-π* transitions associated with the aromatic rings in the polystyrene sulfonate (PSS) structure. This peak confirms the successful incorporation of PSS onto the activated carbon (AC) surface. The absorption in the visible region remains relatively small, which is typical for materials like AC and PSS that do not have significant absorption in this range.

Figure 6b presents Tauc’s graph for determining the band gap energy (E_g_) of the AC@PSS composite. The Tauc plot is constructed by drawing (αhv)^0.5^ against photon energy (hv). The extrapolation of the linear region of the plot to the photon energy axis gives the band gap energy. The band gap value of AC@PSS was determined using Tauc’s Equation (3) as follows:

(αhυ)^1⁄n^ = A (hυ − E_g_) (3)

where α, h, and υ refer to the absorption coefficient, Planck constant (h), and light frequency, respectively. n is a constant that depends on the type of transition (n = 2 for an indirect bandgap and n = 1/2 for a direct bandgap), and A is a constant independent of energy. For the AC@PSS composite, the band gap energy is found to be approximately 4 eV. This band gap indicates that the AC@PSS composite has a wide band gap, which is suitable for photocatalytic applications. The optical properties derived from these analyses are crucial for understanding the photocatalytic activity of the AC@PSS composite. The band gap energy suggests that the material can effectively absorb light, making it a potential candidate for photocatalytic decomposition of pollutants like MB. The UV–vis absorption and Tauc’s plot confirm that the AC@PSS composite can harness light energy, facilitating the generation of reactive species necessary for effective photo-degradation processes.

### 3.2. Photocatalytic Efficacy

#### 3.2.1. MB Removal (%) in the Absence of Uv-Rich Solar Irradiation

**Figure 7:**
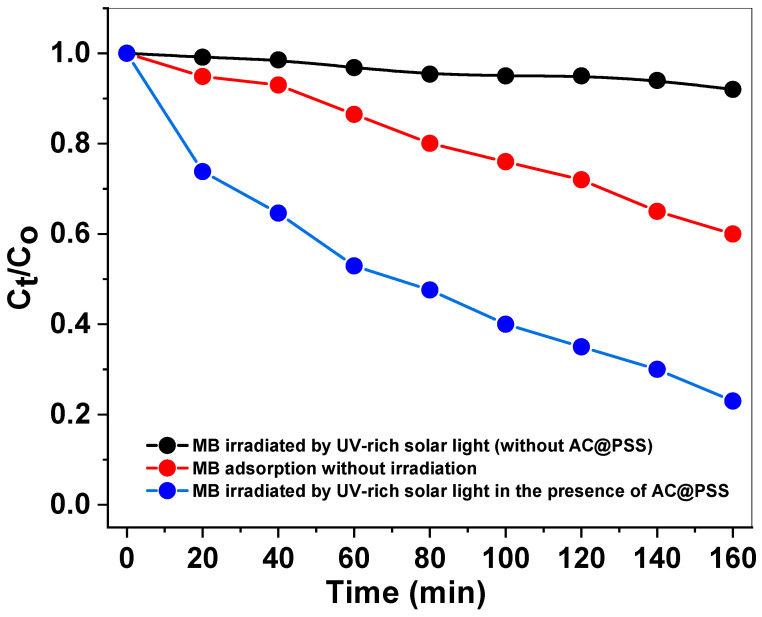
Photocatalytic efficacy (C_t_/C_o_ against time) of MB irradiated by UV-rich solar light (without AC@PSS), MB adsorption without irradiation, and MB irradiated by UV-rich solar light in the presence of AC@PSS.

Figure 7 illustrates the photocatalytic performance of methylene blue (MB) under various conditions by plotting the normalized concentration (C_t_/C_o_) against time. The black curve represents MB irradiated with UV-rich solar light without the presence of AC@PSS, showing minimal degradation over time, which indicates that UV-rich solar light alone has a negligible photocatalytic effect. The red curve, which corresponds to MB adsorption without irradiation, exhibits a slight decrease in C_t_/C_o_, highlighting the adsorption capacity of AC@PSS for MB in the absence of photocatalytic activity. The most significant reduction in C_t_/C_o_ is observed in the blue curve, representing MB irradiated with UV-rich solar light in the presence of AC@PSS. This dramatic decline demonstrates the synergistic effect of photocatalysis and adsorption by AC@PSS, significantly enhancing the degradation efficiency of MB. These results confirm that AC@PSS acts as an effective photocatalyst under UV-rich solar light, enabling superior degradation of MB compared to light irradiation or adsorption alone. The structural and activity correlation of the AC@PSS composite was thoroughly analyzed to understand its enhanced photocatalytic performance. The nitrogen adsorption–desorption isotherms revealed a significant surface area and pore volume, essential for the effective adsorption of methylene blue (MB), thereby facilitating its proximity to reactive sites. The XRD analysis confirmed the amorphous nature of polystyrene sulfonate (PSS) and the structural integrity of activated carbon (AC), while the FTIR results highlighted the successful functionalization of AC with sulfonate groups from PSS. These functional groups play a crucial role in enhancing charge transfer and reducing electron–hole recombination, as evidenced by the improved photocatalytic activity under UV-rich solar light. Additionally, the SEM and TEM images demonstrate the uniform distribution of PSS on the AC surface, which likely contributes to the composite’s synergistic behavior. The combination of these structural features ensures efficient dye adsorption, the generation of reactive oxygen species (ROS), and subsequent degradation of MB, as shown in the kinetic data and scavenger studies. This correlation between the structural properties and photocatalytic activity underscores the efficacy of the AC@PSS composite in advanced dye removal applications.

#### 3.2.2. The Influence of Photocatalytic Parameters on the Degradation of Mb Dye

**Figure 8:**
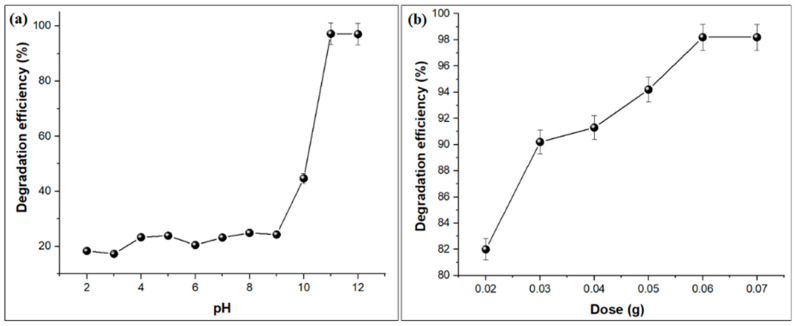
(**a**) Effect of pH and (**b**) catalyst dosage on the photo-degradation efficiency of MB dye using the AC@PSS composite.

The influence of solution pH on the photo-degradation efficiency of MB was studied at various pH values (2–12) of the solution using a constant MB dye concentration (10 ppm) and AC@PSS dosage (60 mg), as presented in Figure 8a. The photo-degradation efficacy of MB dye was slightly boosted from 18.38% to 44.70% with increasing solution pH from 2 to 10 and then increased sharply from pH 10 to 12 with degradation efficiency of 97.19% at pH 11. The results demonstrate that AC@PSS exhibits high degradation efficiency for MB dye in an alkaline medium (pH 11–12) under sunlight irradiation. The photo-degradation efficiency of MB dye improves with increasing pH due to several factors. At higher pH levels, the surface of the AC@PSS composite becomes more negatively charged, enhancing the electrostatic attraction between the negatively charged surface and the positively charged MB dye molecules, facilitating more effective adsorption and degradation. Alkaline conditions also favor the generation of hydroxyl radicals (^•^OH); powerful oxidizing agents are critical in breaking down the dye molecules more efficiently. Additionally, in an alkaline medium, the AC@PSS catalyst may exhibit higher photocatalytic activity due to improved electron–hole separation, preventing recombination and enhancing the overall degradation efficiency. The solubility and stability of the MB dye might also be higher in alkaline conditions, ensuring a more consistent interaction with the catalyst. These factors collectively contribute to the increased photo-degradation efficiency of MB dye with increasing pH, especially in the alkaline range (pH 11–12).

The impact of catalyst dosage on the photo-degradation performance of MB dye was studied at various catalyst dosages from 20 mg to 70 mg using a constant MB dye concentration (10 ppm) at pH: 11 and T: 25 °C, as depicted in Figure 8b. The photo-degradation performance of MB was increased from 82% to 98.20% upon enhancing the catalyst loading from 20 to 60 mg, then it remained constant with further dosage increase to 80 mg. The increases in photo-degradation efficiency of MB with an increase in catalyst dosage are attributed to the availability of a vast number of active centers that enhance the absorption of light, leading to increases in the number of OH radicals developed which can facilitate the photo-degradation of the MB dye [30,31].

#### 3.2.3. Kinetics

**Figure 9:**
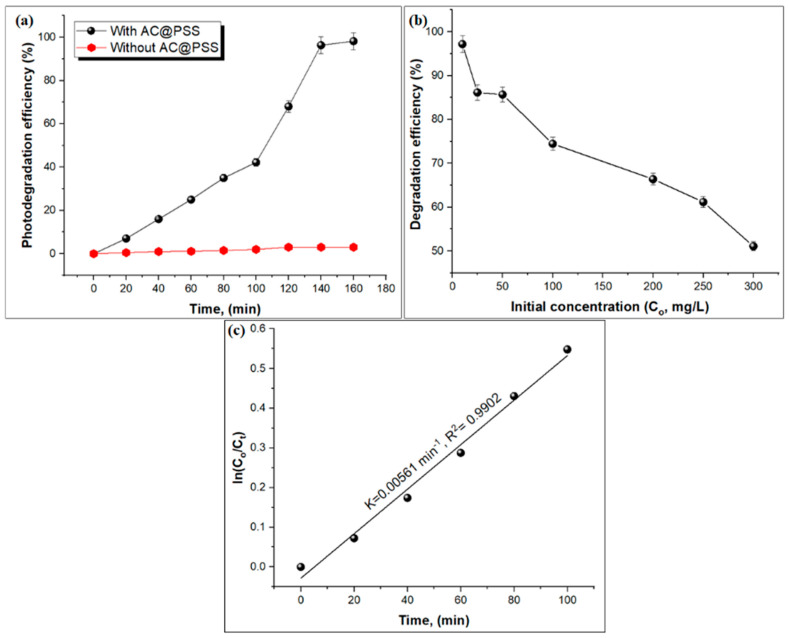
(**a**) The effect of time on the photo-degradation efficiency of MB dye, (**b**) the influence of initial MB concentration on photo-degradation efficiency, and (**c**) photocatalytic degradation kinetics of MB using the AC@PSS composite.

The impact of reaction time on the degradation efficiency of MB dye was examined at various time intervals (20–160 min) using a constant MB dye concentration (10 ppm) at pH: 11 and T: 25 °C, as illustrated in Figure 9a. The photo-degradation efficacy of MB was calculated every 20 min under UV-rich solar irradiation for 160 min. It was observed that the impact of reaction time on the degradation of MB without the AC@PSS composite showed no increase in percentage degradation, showing that MB dye is resistant to UV-rich solar irradiation without the AC@PSS composite. Similar results were observed in a previously reported study [32,33,34]. In addition, no degradation of MB dye was noted in the dark even after 90 min, indicating that the removal of MB is degradation rather than uptake. The degradation efficiency of MB dye gradually increased from 7% to 96.32% with a rising contact time from 20 to 140 min under UV-rich solar irradiation, respectively, and then reached an equilibrium time (98%) at 160 min. The results show that AC@PSS exhibits greater effectiveness in degrading colorants and can achieve sufficient degradation effects within 160 min. The impact of the initial concentration of MB dye on the photo-degradation efficiency of MB dye was studied at different concentrations (10–300 mg/L) using a catalyst dose of 60 mg at pH: 11 and T: 25 °C, as presented in Figure 9b. As the concentration of MB dye solution increased, the photo-degradation performance of MB dye decreased. The maximum photo-degradation efficiency was 97.18% and 86.16% at initial MB concentrations of 10 and 25 mg/L, respectively. As the content of MB dye rises, more MB dye is loaded on the surface of the AC@PSS composite [35]. In a comparison of the present study with several reported studies on the photo-degradation of MB dye, as shown in Table 1, the photocatalytic activity of the prepared AC@PSS composite shows excellent photocatalytic activity when compared to other previous studies.

The kinetics of the photo-degradation rate of MB dye were calculated by employing the Langmuir–Hinshelwood kinetics Equation (4), as follows:

(4) $$ln\;\frac{C_{o}}{C_{t}}=K_{app}\;t\;$$

where C_o_ and C_t_ (mg/L) refer to the initial and remaining concentration of methylene blue dye in solution, respectively. K_app_ refers to the first-order rate constant (min^−1^). The graph (Figure 9c) illustrates the kinetics of the MB photo-degradation process utilizing the AC@PSS composite. The plot of ln(C_o_/C_t_) versus time shows a linear relationship, indicating that the photo-degradation follows first-order kinetics. The rate constant (k) for the decomposition process is estimated to be 0.00561 min^−1^, and the correlation coefficient (R^2^) is 0.9902, suggesting a robust linear fit and the high reliability of the kinetic model. This means that the rate of MB degradation is proportional to the content of the dye, and the AC@PSS composite exhibits effective catalytic activity under the tested conditions. The high R^2^ value further confirms the accuracy of the kinetic data, demonstrating the efficiency of the AC@PSS in degrading MB dye in aqueous solutions.

#### 3.2.4. Investigation of Charge Carrier Trapping and Oxidizing Species

**Figure 10:**
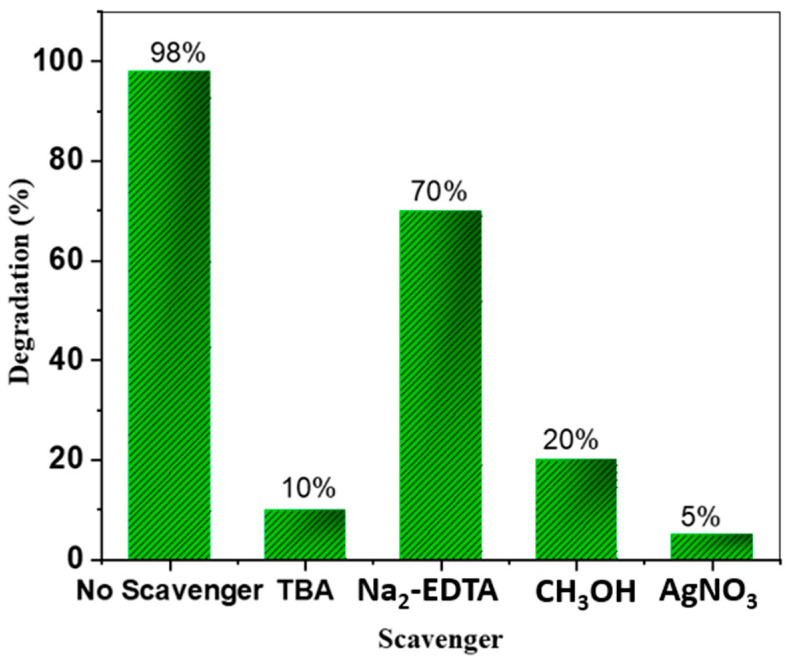
Photocatalytic degradation of MB by the AC@PSS composite in the presence of EDTA as a (h^+^) scavenger, silver nitrate as an (e^–^) scavenger, tert-butyl alcohol as an (^•^OH) scavenger, and methyl alcohol as an (^•^O_2_) scavenger.

Figure 10 illustrates the effect of different scavengers on the photocatalytic degradation of methylene blue (MB) using the AC@PSS composite. The composite achieved 98% degradation without any scavengers, indicating strong photocatalytic performance under standard conditions. However, the introduction of specific scavengers significantly reduced degradation, highlighting the involvement of various reactive species in the process. Degradation dropped to 10% with tert-butyl alcohol (TBA), a hydroxyl radical (^•^OH) scavenger, indicating that hydroxyl radicals play a critical role in the photo-degradation mechanism. The use of Na_2_-EDTA, a hole (h^+^) scavenger, reduced degradation to 70%, suggesting that photogenerated holes also contribute to the process but to a lesser extent. Methanol (CH_3_OH), which scavenges superoxide radicals (O_2_^−•^), lowered degradation to 20%, demonstrating that these radicals have a secondary but notable role. The most significant reduction occurred with silver nitrate (AgNO_3_), an electron (e^−^) scavenger, bringing the degradation down to 5%, underscoring the importance of photogenerated electrons. This suppression occurs because photogenerated electrons play a crucial role in forming reactive species, such as superoxide radicals (^•^O_2_^−^), which are formed through the reduction of oxygen. In the absence of these electrons, the generation of these oxidative species is significantly hindered, leading to a dramatic reduction in degradation efficiency [41]. These findings confirm that the photocatalytic mechanism of MB degradation by the AC@PSS composite is primarily driven by hydroxyl radicals, with substantial contributions from electrons and holes and some involvement of superoxide radicals.

#### 3.2.5. Photocatalytic Degradation Mechanism

**Figure 11:**
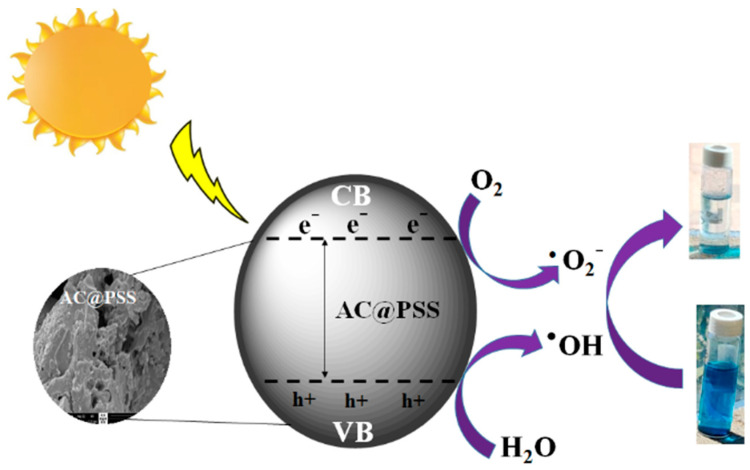
A possible mechanism of the photocatalytic degradation of the MB dyes onto the AC@PSS composite.

The photocatalytic mechanism of the degradation of MB dye using AC@PSS under UV-rich solar irradiation is shown in Figure 11. When photons that are irradiated from UV-rich solar irradiation with energy (4.0 EV) hit the surface of AC@PSS, electrons transition from the VB to the CB, which produces electron–hole pairs. The electrons are captured by dissolved oxygen, producing superoxide radicals (^•^O_2_^−^), which oxidize MB. Moreover, the holes (h^+^) at the VB of AC@PSS reacted with H_2_O, and ^•^OH radicals were produced, which can oxidize MB to produce H_2_O and CO_2_. Therefore, the photo-degradation of MB is caused by the photo-produced radical ^•^OH. Furthermore, the existence of adsorbed oxygen prevents the electrons from recombining with h^+^, enabling water molecules to interact with the free holes, thereby enhancing the efficiency of the photocatalytic process [41]. Moreover, the reactive oxygen species facilitate the oxidative degradation of MB into smaller intermediates and ultimately into CO_2_, water, and mineral byproducts as follows [42,43]:

R + ^•^OH→R^•^ + H_2_O (5)

R + h^+^→R^+^→degradtion product (6)

RCOO^−^ + h^+^→R + CO_2_ (7)

## 4. Conclusions

In summary, we have demonstrated that polystyrene sulfonate-modified activated carbon (AC@PSS) effectively photo-degrades MB dye from aqueous solutions under UV-rich solar irradiation. Characterization through UV-Vis, XRD, SEM, TGA, and FTIR confirmed the formation of polystyrene sulfonate on activated carbon. According to Tauc’s equation, the band gap value of AC@PSS is 4.0 eV. Key parameters for MB dye degradation were identified as pH, catalytic dosage, reaction time, and initial MB concentration. Photocatalytic experiments revealed that AC@PSS achieved 98% degradation efficiency for MB dye in 120 to 160 min at pH 11, a temperature of 25 °C, a catalyst dose of 60 mg, and an initial MB concentration of 10 mg/L. The high photocatalytic performance is attributed to the effective separation of photogenerated electron holes facilitated by activated carbon. This study conclusively demonstrates that AC@PSS is an effective photocatalyst for the photo-degradation of MB molecules.

## Figures and Tables

**Table 1:** Comparison of the photocatalytic activity of the prepared AC@PSS with previous studies.

Prepared Materials	Concentration of MB Dye	Light Source	Degradation Time (min)	Degradation Efficiency (%)	Ref.
ZnO/activated carbon	20	UV–Visible	90	50.21	[36]
CTS-SnO_2_-MWCNTs	3	UV light	180	97.59%	[37]
NaOH/activation carbon	50	UV–Visible	150	74%	[27]
H_3_PO_4_ activated carbon	50	UV–Visible	150	83%	[27]
F-MWCNTs	10	UV light	60	90%	[38]
MWCNT/ZnO	10	UV light	30	94.14%	[39]
Polyacrylonitrile/MWCNTs	10	UV light	120		[40]
**AC@PSS**	10	UV-rich solar irradiation	160	98%	**This study**

## Data Availability

The original contributions presented in this study are included in the article; further inquiries can be directed to the corresponding author.
